# Expression of full-length p53 and its isoform Δp53 in breast carcinomas in relation to mutation status and clinical parameters

**DOI:** 10.1186/1476-4598-5-47

**Published:** 2006-10-20

**Authors:** Lars O Baumbusch, Simen Myhre, Anita Langerød, Anna Bergamaschi, Stephanie B Geisler, Per E Lønning, Wolfgang Deppert, Irene Dornreiter, Anne-Lise Børresen-Dale

**Affiliations:** 1 Department of Genetics, Institute for Cancer Research, Rikshospitalet-Radiumhospitalet Medical Center, 0310 Oslo, Norway; 2 Department of Medicine, Section of Oncology, Haukeland University Hospital, 5021 Bergen, Norway; 3 Heinrich-Pette-Institut für Experimentelle Virologie und Immunologie an der Universität Hamburg, Martinistr. 52, 20251 Hamburg, Germany; 4 Medical Faculty, University of Oslo, Oslo, Norway

## Abstract

**Background:**

The tumor suppressor gene *p53 (TP53) *controls numerous signaling pathways and is frequently mutated in human cancers. Novel p53 isoforms suggest alternative splicing as a regulatory feature of p53 activity.

**Results:**

In this study we have analyzed mRNA expression of both wild-type and mutated p53 and its respective Δp53 isoform in 88 tumor samples from breast cancer in relation to clinical parameters and molecular subgroups. Three-dimensional structure differences for the novel internally deleted p53 isoform Δp53 have been predicted. We confirmed the expression of Δp53 mRNA in tumors using quantitative real-time PCR technique. The mRNA expression levels of the two isoforms were strongly correlated in both wild-type and *p53*-mutated tumors, with the level of the Δp53 isoform being approximately 1/3 of that of the full-length p53 mRNA. Patients expressing mutated full-length p53 and non-mutated (wild-type) Δp53, "mutational hybrids", showed a slightly higher frequency of patients with distant metastasis at time of diagnosis compared to other patients with p53 mutations, but otherwise did not differ significantly in any other clinical parameter. Interestingly, the p53 wild-type tumors showed a wide range of mRNA expression of both p53 isoforms. Tumors with mRNA expression levels in the upper or lower quartile were significantly associated with grade and molecular subtypes. In tumors with missense or in frame mutations the mRNA expression levels of both isoforms were significantly elevated, and in tumors with nonsense, frame shift or splice mutations the mRNA levels were significantly reduced compared to those expressing wild-type p53.

**Conclusion:**

Expression of p53 is accompanied by the functionally different isoform Δp53 at the mRNA level in cell lines and human breast tumors. Investigations of "mutational hybrid" patients highlighted that wild-type Δp53 does not compensates for mutated p53, but rather may be associated with a worse prognosis. In tumors, both isoforms show strong correlations in different mutation-dependent mRNA expression patterns.

## Background

The tumor suppressor and transcription factor p53 (TP53) is a key regulator of cell integrity with impact on cell cycling, growth, DNA repair, cell cycle arrest, or apoptosis (see reviews [1-4]). Correct p53 signaling is essential for preventing tumor growth (see reviews [5-7]). The structure of the TP53 protein has been studied extensively and different conserved domains have been identified [8,9]: the transcription activation domain, the sequence-specific DNA-binding domain with a subdomain interacting with the 53bp2 SH3 domain, a non-structured spacer region containing a bipartite nuclear localization signal, a tetramerization domain with a nuclear export signal subdomain, and a C-terminal domain modulating DNA-binding [10-12]. The central core domain of p53 is built of highly conserved anti-parallel beta-sheet scaffolds assembling two alpha-helical loops interacting with the grooves in the DNA [13]. The functional unit of p53 is a tetramer, where the C-terminal ends of two carboxyl-terminal peptides form a dimer, and two dimers assemble to tetramers [14,15].

Several p53 isoforms have been described, but for most of them knowledge has been restricted due to unclear function, their expression only at certain conditions or at very low levels, or their detection in other organisms than humans (see reviews [16,17]). Initially, human p53 was shown to have only one promoter and two alternative splice forms, p53i9 [18] and Δ40p53 [19-21]. Commonly p53 alternative splice forms diverge from full-length p53 by altering the N-terminal [19,20,22] or the or the C-terminal domains [18,23], but preserve the central domain. Recently, a new internal promoter together with four additional N- and C-terminal isoforms were found [22], and the first internal splice form Δp53 was discovered [24]. The novel alternative splice form Δp53 is unique due to its unusual splice sites and expression pattern. In addition, its activation profile differs from that of p53 [24]. The importance of regulatory features of p53 isoforms has likely been underestimated [16], in particular, whether mutations in the *p53 *gene in tumors have different effect on the various isoforms. The various functions associated with the novel p53 alternative splice forms have attracted attention and opened questions about possible other functions (see comments [17,25]), since differential expression of p53 isoforms represents an interesting option for promoter selectivity, tissue-specific activation, and selective activation of downstream targeting genes.

The *p53 *gene has the highest mutation frequency in human tumors [26,27], with large varieties in the positions of the alterations and in the mutation spectra due to environmental, geographical, racial and other factors [28-31]. Mutations in the *p53 *gene are found in 20–30% of breast carcinomas (see reviews [3,28]), most of them being missense point mutations mainly located in or close to the conserved DNA-binding region [32]. The p53 mutation status has been shown to be an independent prognostic marker for poor outcome in breast cancer [33,34]. All mutations are "loss-of-function" regarding the tumor suppressor functions of wild-type p53, but some reports also describe that at least some mutations exert a novel "gain of function" (see review [35]).

In this paper we have studied mRNA expression of full-length p53 and its Δp53 isoform in both *p53 *wild-type and mutant tumors from 88 breast cancer patients. We used quantitative real-time PCR (qRT-PCR) and related the mRNA expression levels to clinical and biological data. We wanted to explore whether patients with mutations affecting both full-length and Δp53 differ with respect to clinical and molecular markers from patients with mutations affecting only the full-length and not the Δp53 isoform.

## Results

### Bioinformatic analyses of exon transition, structural domain organization, and prediction of three-dimensional protein folding for the alternative splice form Δp53

**Figure 1:**
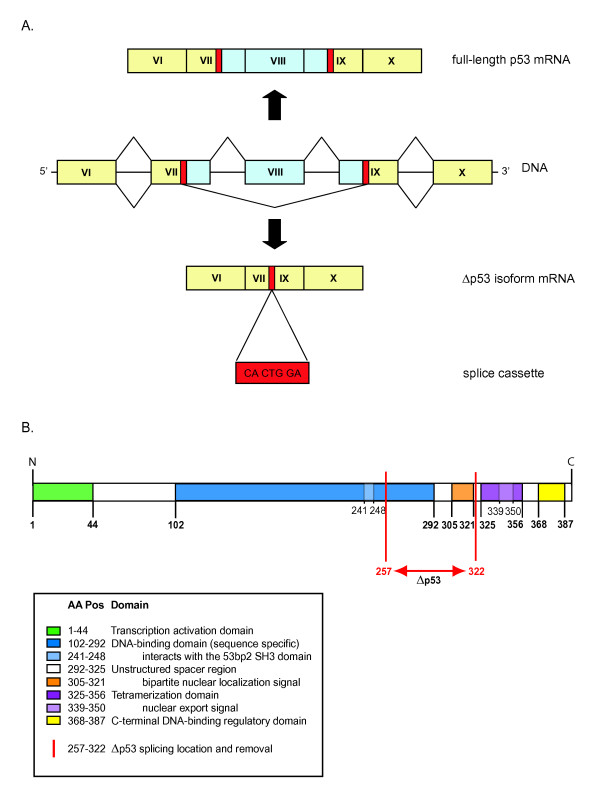
**Schematic representation of the full-length p53 and the alternative splice form Δp53**. (A) The mRNA structure of exons VI to X of the full-length *p53 *and the *Δp53 *isoform are shown. The removed sequence in Δp53 is located in parts of exon VII, in exon VIII, and in a fraction of exon IX. The alternative splice cassette junction sequence, represented twice in the full-length p53 and once in the alternative splice form, is indicated in red. (B) Structural organization of the full-length p53 and Δp53 and its functional domains. p53 protein domain classification and their locations along the protein according to Swiss-Prot/TrEMBL [12]. Subdomains of main structures are indicated with various colors. Red lines mark the part eliminated by the splicing process of Δp53 and covering aa 257 to 322 of the DNA-binding domain and the complete non-structured spacer region with bipartite nuclear localization signal.

Δp53 is a novel alternative splice form that differs from the full-length p53 form by lacking parts of exon 7, complete elimination of exon 8 and partial removal of exon 9 [24]. The uncommon splice mechanism involves two 7 base pair long cassettes with an identical sequence in exon 7 and exon 9, of which one is retained in the isoform (Figure 1A; for sequence details see Additional file 1). We estimated the splice site plausibility by analyzing the cassette sequence for exonic splicing enhancer (ESE) motifs using the ESEfinder [36]. Exonic enhancers are potential binding sites for splicing factors of the highly conserved serine/arginine-rich (SR) protein family. The cassette motif gives a high score (3.5) for the splice factor SF2/ASF protein and has some similarity with known signal sequences for alternative splicing (see review [37]). According to Swiss-Prot/TrEMBL structural protein domain classification [12] full-length p53 consists of a transcription activation domain (aa 1–44), a DNA-binding domain (aa 102–292), an unstructured spacer containing a bipartite nuclear localization signal (aa 305–321; a bipartite nuclear localization signal domain is defined as two adjacent basic amino acids with a spacer region of any 10 residue and at least three basic residues (Arg or Lys) in the five positions following the spacer region [38]), a tetramerization domain (aa 325–356), and a C-terminal regulatory domain (aa 368–387) (Figure 1B). In Δp53, this domain organization is modified by the removal of 66 amino acid (residues 257 to 322), which mainly disturbs the DNA-binding domain, and eliminates the spacer with the bipartite nuclear localization signal. The DNA-binding domain (aa 102–292) is truncated, but the 53bp2 SH3 domain remains intact, while the spacer with the bipartite nuclear localization signal domain (aa 305–321) is entirely removed.

**Figure 2:**
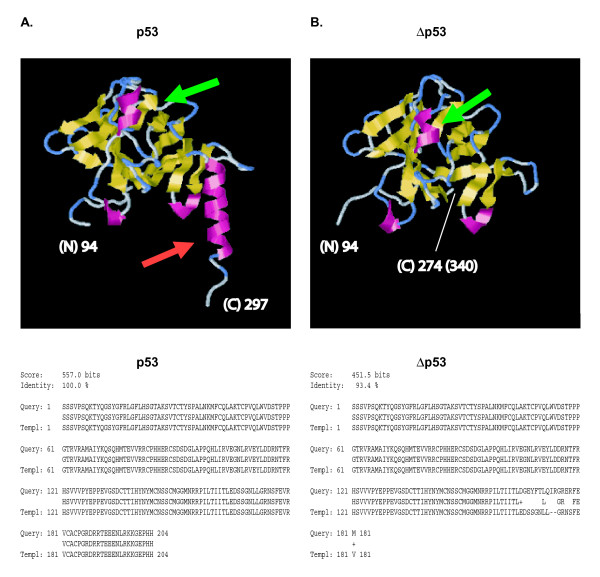
**Predicted structural organization of the p53 and Δp53 core domains illustrated by three-dimensional models**. Illustrated are CPH predicted models [39] of the p53 and Δp53 isoforms using the RasMol program. The p53 core domain of 204 aa (total length of wild-type p53 is 393 aa) is predicted from aa 94 to 297 with a prediction identity of 100% (557.0 bits score) and the Δp53 core domain is predicted from aa 94 to 274 (total length of Δp53 is 327 aa) with a 93.4% identity and 451.5 bits score (protein position 274 in the Δp53 accordingly corresponds to protein position 340 in the full-length p53). Models and prediction structures for p53 (A) and Δp53 (B) are shown, and the variations are colored by secondary structures as follows: alpha helices in magenta, beta sheets in yellow, turns in pale blue, and all other residues are colored white. Differences between the isoform predictions are indicated with arrows and the N-terminal starting and C-terminal end points are marked in the figure. Due to the alternative splicing a major alpha-helical structure is missing in Δp53 (in A red arrow) and the tertiary protein structure of Δp53 is slightly more compact, as can bee seen from the moved alpha helix, indicated by green arrows. Below: uploaded protein core for the three-dimensional structure prediction query and received structural template.

To elucidate the differences in three-dimensional structure between p53 and Δp53 the complete sequences of p53 and Δp53 were submitted to predictive CPH-modelling using the web-based service of the Technical University of Denmark [39]. The core domains of the proteins were modelled based on available structures in the database: p53 was predicted from aa 94 to 297 with an identity of 100% (557.0 bits score) and Δp53 from aa 94 to 274 with 93.4% identity by a 451.5 bits score. (The removal of residues 257 to 322 by the Δp53 specific splice process changes the position numbers of the Δp53 predicted protein relative to the full-length protein: residue 274 in Δp53 corresponds to 340 in the full-length p53 protein). The isoforms clearly vary in both their secondary and their calculated three-dimensional structure, even though the prediction identity for Δp53 was limited. The three-dimensional structure prediction, illustrated by using the RasMol program (Version 2.7.2.1.1.), reveals a modified structure for Δp53 in comparison to p53, resulting in a position shift of an alpha-helical structure and thus condensing the structure (Figure 2). In Δp53 a major alpha-helical structure at the C-terminal end is deleted, altering its further orientation. The structural data are supported by functional studies in cell lines, where the isoforms have different transactivation activities for the *p21, mdm2, 14-3-3-sigma, bax *and *PIG3 *promoters [24].

### p53 and Δp53 mutational status in relation to clinical, pathological and biological factors

**Figure 3:**
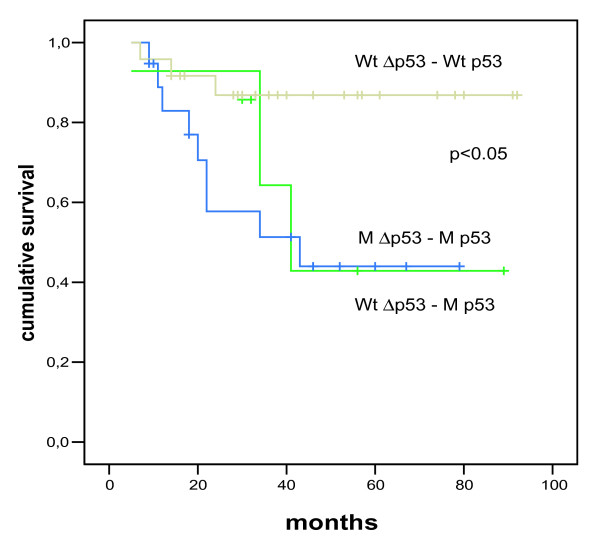
**Kaplan-Meier plot of survival rates for patientswith mutated and unmutated full-length p53 and Δp53**. Cumulative breast cancer survival for a subset of patients (50) is shown for three groups of patients, depending on their mutational status of p53 versus Δp53: patients with wild-type Δp53 and wild-type p53 (Wt Δp53 – Wt p53; n = 24), "mutational hybrid" patients with non-mutated (wild-type) Δp53 and mutated full-length p53 (Wt Δp53 - M p53; n = 7), and patients with mutations in Δp53 and p53 (M Δp53 – M p53; n = 19); the significance is *p *= 0.0498.

We analyzed the existence and expression of the Δp53 isoform in breast tumors from patients with advanced disease at the mRNA level and asked whether mutations present in both isoforms had different effects on clinical and molecular parameters compared to mutations found only in the full-length form. These patients have previously been analyzed by whole genome expression microarrays and grouped according to their expression profile ([40-43] for cohorts A, B and D, and unpublished results for cohort C). Gel electrophoresis followed by qRT-PCR confirmed that Δp53 together with full-length p53 was present in all tumor samples. Patients with tumors harbouring mutations residing inside the spliced out region of the Δp53 isoform represented the rare mutational genotype of mutated full-length p53 and wild-type Δp53 and were termed "mutational hybrids" (Wild type Δp53/Mutant p53 = WtM). These patients where grouped (11 patients) and compared to patients with mutations before and after the spliced region affecting both isoforms (27 patients), and to patients without mutations (50 patients). Of the 27 tumors with mutations affecting both isoforms 14 were missense mutations, two were in frame mutations, one a nonsense mutation, four were splice mutations, and six had frame shift mutations. (For a full description of the various mutations see Additional file 2). Based on the mutation type the p53/Δp53 double mutant group (MM) was further subdivided into the MI group with missense and in frame mutations, and the MII group with nonsense, frame shift and splice mutations (Table 1). Kruskal Wallis rank tests were performed for differences in clinical and molecular parameters in three (WtWt-WtM-MM) or four classes (WtWt-WtM-MIMI-MIIMII) and the Mann-Whitney test was used to test for independent association between subgroups. A slightly higher frequency of patients with distant metastasis at time of diagnosis was observed in the WtM group compared to the MIMI group (*p *< 0.07). No other significant differences were observed for the "mutational hybrid" group weighted against the other groups. We analyzed whether the "mutational hybrid" genotype had an effect on patient survival time in a subset of patients from two prospective studies [44,45] of comparable treatment and uniformity. In the Kaplan-Meier plot (Figure 3) survival data for a total of 50 patients without distant metastases at time of diagnosis are shown. The survival rates in patients with the "mutational hybrid" genotype (Wt Δp53 – M p53) was similar to the survival rate in patients with mutations in both p53 and Δp53 (M Δp53 – M p53), and the survival rates in these two groups were significantly different compared to patients with wild-type Δp53 and p53 (Wt Δp53 – Wt p53) (*p *< 0.05).

**Table 1:** Relationship between p53 and Δp53 mutation status and their correlation to biological and clinical factors

			Wild type (Wt) Wt Δp53 Wt p53		Mutational hybrid Wt Δp53 M p53		Mutations (MI = MS, IF) MI Δp53 MI p53		Mutations (MII = NS, FS, SP, SC) MII Δp53 MII p53			
								
Characteristic	Total No.		No. of patients	(%)	No. of patients	(%)	No. of patients	(%)	No. of patients	(%)	Groups	*p*
**Lymph node status**	**88**	Node-negative	**16**	32.0	**1**	9.1	**4**	23.5	**6**	60.0	**WtWt-WtM^1^**	**n.s.**
		Node-positive	**34**	68.0	**10**	90.9	**13**	76.5	**4**	40.0	**WtM-MIMI^1^**	**n.s.**
											**WtM-MM^1^**	**n.s. (*p *< 0.09)**
											**WtWt-WtM-MM^2^**	**n.s.**
											**WtWt-WtM-MIMI-MIIMII^2^**	**n.s. (*p *< 0.08)**
												
**Estrogen receptor status**	**88**	Negative	**10**	20.0	**3**	27.3	**8**	47.1	**3**	30.0	**WtWt-WtM^1^**	**n.s.**
		Positive	**40**	80.0	**8**	72.7	**9**	52.9	**7**	70.0	**WtM-MIMI^1^**	**n.s.**
											**WtM-MM^1^**	**n.s.**
											**WtWt-WtM-MM^2^**	**n.s.**
											**WtWt-WtM-MIMI-MIIMII^2^**	**n.s.**
												
**Progesterone receptor status**	**88**	Negative	**13**	26.0	**4**	36.4	**9**	52.9	**6**	60.0	**WtWt-WtM^1^**	**n.s.**
		Positive	**37**	74.0	**7**	63.6	**8**	47.1	**4**	40.0	**WtM-MIMI^1^**	**n.s.**
											**WtM-MM^1^**	**n.s.**
											**WtWt-WtM-MM^2^**	***p *< 0.04**
											**WtWt-WtM-MIMI-MIIMII^2^**	**n.s. (*p *< 0.09)**
												
**ERBB2/HER status**	**60**	Negative	**29**	80.6	**5**	62.5	**10**	90.9	**1**	20.0	**WtWt-WtM^1^**	**n.s.**
		Positive	**7**	19.4	**3**	37.5	**1**	9.1	**4**	80.0	**WtM-MIMI^1^**	**n.s.**
											**WtM-MM^1^**	**n.s.**
											**WtWt-WtM-MM^2^**	**n.s.**
											**WtWt-WtM-MIMI-MIIMII^2^**	***p *< 0.02**
												
**Distant metastisis at time of diagnosis**	**65**	Negative	**30**	90.9	**7**	77.8	**15**	100.0	**7**	87.5	**WtWt-WtM^1^**	**n.s.**
		Positive	**3**	9.1	**2**	22.2	**0**	0.0	**1**	12.5	**WtM-MIMI^1^**	**n.s. (*p *< 0.07)**
											**WtM-MM^1^**	**n.s.**
											**WtWt-WtM-MM^2^**	**n.s.**
											**WtWt-WtM-MIMI-MIIMII^2^**	**n.s.**
												
**Grade**	**88**	1	**5**	10.0	**0**	0.0	**0**	0.0	**0**	0.0	**WtWt-WtM^1^**	**n.s.**
		2	**24**	48.0	**4**	36.4	**4**	23.5	**4**	40.0	**WtM-MIMI^1^**	**n.s.**
		3	**21**	42.0	**7**	63.6	**13**	76.5	**6**	60.0	**WtM-MM^1^**	**n.s.**
											**WtWt-WtM-MM^2^**	***p *< 0.03**
											**WtWt-WtM-MIMI-MIIMII^2^**	***p *< 0.05**
												
**Response to chemotherapy**	**54**	Response	**24**	92.3	**8**	88.9	**9**	81.8	**5**	62.5	**WtWt-WtM^1^**	**n.s.**
		None response	**2**	7.7	**1**	11.1	**2**	18.2	**3**	37.5	**WtM-MIMI^1^**	**n.s.**
											**WtM-MM^1^**	**n.s.**
											**WtWt-WtM-MM^2^**	**n.s.**
											**WtWt-WtM-MIMI-MIIMII^2^**	**n.s.**
												
Subgroups*	**84**	Luminal A	**25**	53.2	**2**	18.2	**2**	12.5	**0**	0.0	**WtWt-WtM^1^**	**n.s. (*p *< 0.08)**
		Luminal B	**6**	12.8	**2**	18.2	**4**	25.0	**3**	30.0	**WtM-MIMI^1^**	**n.s.**
		ERBB2	**7**	14.9	**3**	27.3	**4**	25.0	**4**	40.0	**WtM-MM^1^**	**n.s.**
		Basal	**4**	8.5	**4**	36.4	**6**	37.5	**2**	20.0	**WtWt-WtM-MM^2^**	***p *< 0.008**
		Normal-like	**5**	10.6	**0**	0.0	**0**	0.0	**1**	10.0	**WtWt-WtM-MIMI-MIIMII^2^**	***p *< 0.02**

MS = missense mutations, NS = nonsense mutations, FS = frame shift mutations, IF = in frame mutations, SP = splice mutations, SC = Δp53 splice cassette mutations

M = MI + MII

^1 ^for WtWt-WtM, WtM-MIMI and WtM-(MIMI+MIIMII) the Mann-Whitney test was performed

^2 ^for WtWt-WtM-(MIMI+MIIMII) and WtWt-WtM-MIMI-MIIMII the Kruskal Wallis test was performed

* The tumors have previously been subjected to whole genome microarray analysis and classified into subgroups according to their expression profile (see Material and Methods)

### qRT-PCR analysis of p53 and Δp53 mRNA expression levels using Universal Human Reference cell lines, mutated, and non-mutated human breast tumors

**Figure 4:**
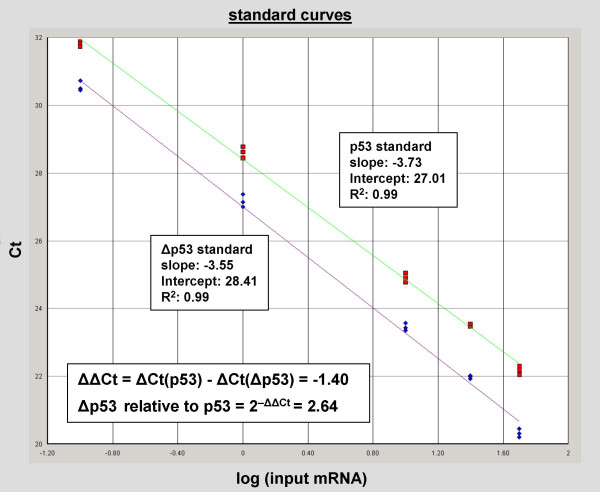
**p53 and Δp53 standard curve by qRT-PCR**. Standard curve plotting showing CO (concentration) in log scale *versus *Ct (Cycle threshold). The fluorescence signal of the reporter dye (*FAM*) subtracted by the baseline signal of the passive reference dye (ROX) results in a ratio defined as the normalized reporter signal ΔRn. ΔRn increases with accumulating PCR cycles until it reaches a plateau. Ct represents the fractional cycle number at which significant increase in Rn above a baseline signal of the passive reference dye (ROX) can be detected. Standard curve points are based on serially diluted cDNAs of a mixture of 10 human cancer cell lines in a 1,5 orders of linear dynamic range. All samples were performed in triplets. *Red quadrates *illustrate data for p53 standard curve; *blue squares *show data for Δp53 standard curve of the same template. The red line linear represents regression of the standard quantity and the C_T _value for Δp53 and green line stand for linear regression of the standard quantity and the C_T _value for p53. The comparative Ct value between the two standard curves is 1.40.

Using qRT-PCR, the expression level of both full-length p53 and Δp53 mRNA were determined. Both isoforms were present in the Universal Human Reference of 10 human cell lines mixture and in the human breast tumor samples. The p53 and Δp53 mRNA levels were determined by standard curve measurements in a 1.5 orders of linear dynamic dilution range performed on the same plate. Both splice forms showed similar slopes and accordingly have equivalent target efficiencies (Figure 4). Under the presupposition of equivalent efficiencies the Comparative Ct (Cycle threshold) ΔΔCt method can be selected to compare normalized expression levels of different samples relative to a calibrator sample.

Using the comparative Ct method we compared the linearized (2^-ΔΔCt^) expression levels of the standard curves of the two alternative splice forms relative to each other, whereas for the various tumor samples the more robust standard curve method was applied. An investigation of different housekeeping genes revealed two independent genes, PMM1 and RPL32 [46], suitable for mRNA expression level determination in human breast tumors (for details see Material and Methods). Repeated experiments showed that standard curves for full-length p53 mRNA intercept at 27.01, while Δp53 had a Ct intercepting at 28.41 (Figure 4). The comparative Ct value ΔΔCt is given as the Ct intercept difference between the two isoform standard curves for ΔCt(p53) = 27.01 and ΔCt(Δp53) = 28.41, with a value of -1.40. Accordingly, Δp53 is expressed 2.64 times lower (2^-ΔΔCt ^= 2.64) relative to the full-length p53 isoform.

**Figure 5:**
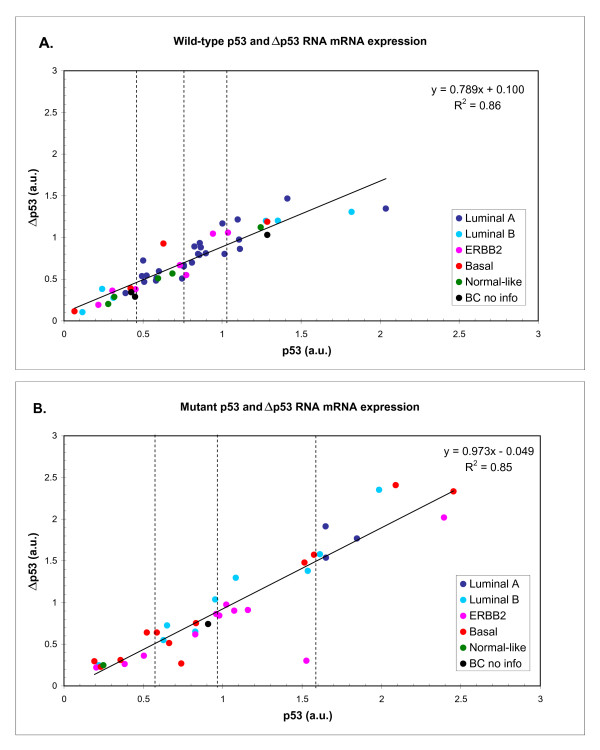
**Correlation between mRNA expression level of full-length p53 and Δp53 in relation to different molecular breast cancer subtypes in A. wild-type p53 tumors or B. p53-mutated tumors**. Both wild-type samples (A) and mutated samples (B) show a wide range of mRNA expression in a.u. (arbitrary units) with significant association to molecular breast cancer subtypes. Note that the spreading is different in the two groups with a more continuously spreading in the wild-type tumors compared to the mutated onces. Samples are marked by their molecular subtype characteristics: Luminal A (dark blue), Luminal B (light blue), ERBB2 (red), Basal (pink), Normal-like (green) and without information (black). Horizontal lines illustrate borders between the quartiles for wild-type (25% = 0.452; 50% = 0.754 and 75% = 1.022) or mutants (25% = 0.569; 50% = 0.956 and 75% = 1.584). The regression line for all samples is drawn with an equation y = 0.789x + 0.100 and a regression coefficient of 0.86 for wild-type and y = 0.973x - 0.049 and a regression coefficient of 0.85 for the mutant samples.

**Figure 6:**
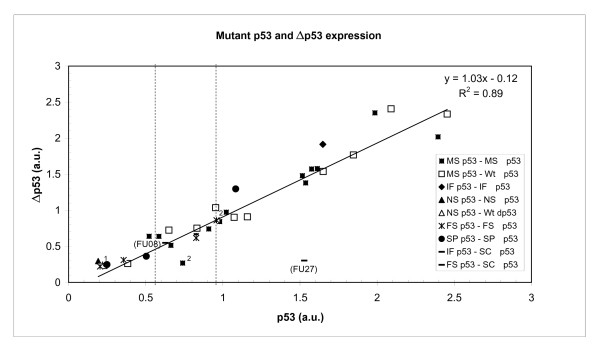
**Correlation between mRNA expression level of full-length p53 versus Δp53 in breast carcinomas with various p53 mutations**. p53 and Δp53 mRNA expression levels of mutated p53 in human breast tumors are shown with p53 relative mRNA expression in a.u. (arbitrary units) on x-axis and Δp53 on y-axis. Different mutation types are indicated by various symbols. "Mutational hybrids", mutations represented on full-length p53, but removed in Δp53, are marked with open symbols. Mutations present in both isoforms are specified with filled symbols. The shape of the symbols indicate the various mutation types: ■, □ missense mutations, ◊ in frame mutations, ▲, Δ nonsense mutations, * frame shift mutations, ● splice mutations, - mutations in the splice cassette (the two samples full-filling this criteria are highlighted with their sample ID). Horizontal lines show borders between the median values of the relative mRNA expression subgroups: MII vs Wt vs MI. The regression line for missense mutations is drawn with an equation y = 1.03x - 0.12 and a regression coefficient of 0.89. (^1 ^low p53 value in this sample might be due to mutation in p53 primer binding site, ^2 ^low Δp53 value in this sample might be due to mutation in Δp53 primer binding site – for details see Additional file 2).

Comparison of the mRNA levels of the two isoforms p53 and Δp53 in 88 tumors samples revealed a high correlation in both mutated and non-mutated tumors with a correlation coefficient of r^2 ^= 0.86 for wild-type and r^2 ^= 0.85 for mutant tumors, respectively (Figure 5A and 5B). The expression levels of Δp53 mRNA in the two tumor samples with in frame (FU27), or with a frame shift (FU08) mutation located in the splice cassette were very low (Figure 6), further confirming the existence of Δp53 in the other human tumor samples.

**Figure 7:**
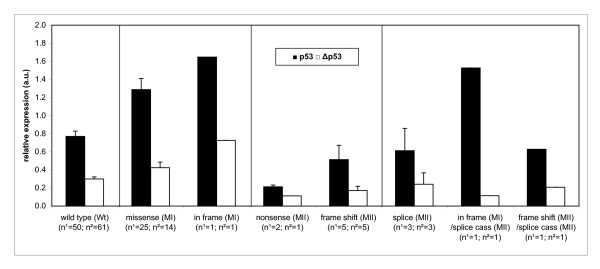
**Histogram of p53 and Δp53 relative mRNA expression**. Mean and SEM of the relative p53 and Δp53 mRNA expression in a.u. (arbitrary units) for the different mutation classes: missense, in frame, nonsense, frame shift, splice or splice cassette (splice cass). p53 and Δp53 mRNA expression levels are proportion adjusted according to the comparative Ct of 2.64. The number of cases (n) is given by ^1 ^for p53 and by ^2 ^for Δp53, respectively. Differences in case numbers for p53 and Δp53 are due to some mutations which are located inside the area, but removed by the alternative splicing process of Δp53. The expression level for both full-length p53 and Δp53 between the various mutational classes were highly significant (Table 2).

Interestingly, the expression level of both mutant and wild-type p53 varied by more than 3-fold for both full-length and Δp53, and the different mutation types showed a large variation in mRNA expression for both isoforms (Figure 6). Tumors with wild-type p53 have an average mRNA expression level of 0.770 arbitrary units (a.u.), tumors with missense or in frame mutations (MI) showed elevated mRNA abundance of 1.310 a.u., while nonsense, frame shift or splice mutations (MII) had lower mRNA levels with an average of 0.496 a.u. (Figure 7 and Table 2). The wild-type p53 isoform mRNA level were significantly different (*p *< 0.00002) from the levels in both mutation groups and the same was the case for the mRNA level of the Δp53 isoforms (*p *< 0.004) (Figure 7 and Table 2).

**Table 2:** p53 or Δp53 relative mRNA expression

	Wild type (Wt)		Mutation group I (MI) missense and in frame mutations		Mutation group II (MII) nonsense, frame shift and splice mutations			
					
	No. of patients	Mean a.u. (± SEM) Median	No. of patients	Mean a.u. (± SEM) Median	No. of patients	Mean a.u. (± SEM) Median	Groups	*p*
**full-length p53**	**50**	0.770 (± 0.059)	**27**	1.310 (± 0.116)	**11**	0.496 (± 0.100)	**Wt-MI-MII^1^**	***p *< 0.00002**
							Wt-MI^2^	*p *< 0.0002
		0.753		1.159		0.357	Wt-MII^2^	*p *< 0.04
							MI-MII^2^	*p *< 0.002
**Δp53**	**61**	0.790 (± 0.061)	**15**	1.171 (± 0.162)	**11**	0.482 (± 0.101)	**Wt-MI-MII^1^**	***p *< 0.004**
							Wt-MI^2^	*p *< 0.04
		0.725		0.974		0.310	Wt-MII^2^	*p *< 0.02
							MI-MII^2^	*p *< 0.002

Mean is given in relative expression in arbitrary units (a.u.)

^1 ^for Wt-MI-MII the Kruskal Wallis test was performed

^2 ^for Wt-MI, WT-MII and MI-MII the Mann-Whitney test was performed

### mRNA expression levels of mutated and non-mutated human p53 and Δp53 in human breast tumors in relation to clinical and biological parameters

The wild-type full-length p53 mRNA expression levels display a wide range. We explored whether mRNA expression levels were associated with particular clinical and/or molecular parameters. Therefore, we divided the wild-type p53 mRNA expression profiles into three classes of quartiles, merging the two midst quartiles to one group: (Q1) <25%, (Q2&Q3) 25–75% and (Q4) >75%. Kruskal Wallis tests were used to analyze for differences between the three groups and the Mann-Whitney test was used for tests for differences between any two groups (Table 3). Molecular breast cancer subtype distribution was significantly different among the various p53 mRNA expression groups (*p *< 0.03). The Luminal A subtype is dominating in the majority of the middle and high expression group, while tumors with low wild-type p53 mRNA expression have a low proportion of Luminal A tumors (9.1%) and a high fraction of the Luminal B and ERBB2 subgroups. Middle quartiles were showing the highest fractions of estrogen receptor positive tumors (96%) compared to only 69% and 62% in the low expression (Q1) and high expression quartiles (Q4), respectively (*p *< 0.03). Similar observations were made for the progesterone receptor status. Low wild-type p53 mRNA expression was significantly associated with grade 3 tumors in Q1, while Q2/Q3 and Q4 in their majority were of grade 2 (*p *< 0.003). No significant association was found for age, menopausal status, lymph node status, ERBB2/HER status, tumor histology, or p53 LOH. For the Δp53 wild-type distribution our analysis of expression quartiles revealed similar associations for biological and clinical parameters (data not shown).

We then looked for the relationship between the various molecular subgroups and mutation classes in both p53 and Δp53 (Table 4). The Luminal A subtype is significantly overrepresented (47%) in patients with wild-type Δp53 tumors compared to patients with MI (13%) or MII (0%) mutations (*p *< 0.04). In tumors with MI type mutations the Basal subgroup was dominating (40%) and in tumors with MII mutations the ERBB2 subtype (46%) was most highly represented (Table 4).

Beside mutational status and despite relatively low numbers of tumor samples, the mRNA expression levels for both p53 and Δp53 mRNA mutant and wild-type demonstrated interesting results: The Luminal A subtype revealed scattered p53 mRNA expression levels in wild-type p53, but rather high mRNA expression in the mutated p53 tumors, while the Luminal B subtype had either a very low or very high mRNA expression rate in the wild-type p53, but normal expression distribution in mutant p53 tumors. The ERBB2 molecular breast cancer subtype indicates low p53 mRNA levels in mutant p53, but normal scattering in wild-type p53 tumor samples (Figure 6).

Association rank tests were performed, scoring wild-type and the different mutation types of the Δp53 isoform in relation to various clinical, pathological and biological factors [see Additional file 3]. In contrast to full-length p53 (Table1), we observed a significant lower fraction of patients with lymph node positive tumors in the Δp53 MII group compared to the Wt and MI group. Δp53 isoform shows associations for the same parameters and similar to the observed associations for full-length p53 [see Additional file 3].

**Table 3:** Relationship between wild-type full-length p53 mRNA expression level and clinical, pathological and biological factors

				wild-type full-length p53 RNA level							
				Q1 (<25%)		Q2 & Q3 (25–75%)		Q4 (>75%)			
								
Characteristic		Total No.	Total (%)	No. of patients	(%)	No. of patients	(%)	No. of patients	(%)	Groups	*p*
**Estrogen receptor status**	Negative	**10**	20	**4**	30.8	**1**	4.2	**5**	38.5	**Q1-Q2/3-Q4^1^**	***p *< 0.03**
	Positive	**40**	80	**9**	69.2	**23**	95.8	**8**	61.5	Q1-Q2/3^2^	*p *< 0.03
										Q1-Q4^2^	n.s.
										Q2/3-Q4^2^	*p *< 0.009
											
**Progesterone receptor status**	Negative	**13**	26	**3**	23.1	**3**	12.5	**7**	53.8	**Q1-Q2/3-Q4^1^**	***p *< 0.03**
	Positive	**37**	74	**10**	76.9	**21**	87.5	**6**	46.2	Q1-Q2/3^2^	n.s.
										Q1-Q4^2^	n.s.
										Q2/3-Q4^2^	*p *< 0.009
											
**ERBB2/HER status**	Negative	**29**	80.6	**9**	75.0	**13**	86.7	**7**	77.8	**Q1-Q2/3-Q4^1^**	**n.s.**
	Positive	**7**	19.4	**3**	25.0	**2**	13.3	**2**	22.2	Q1-Q2/3^2^	n.s.
										Q1-Q4^2^	n.s.
										Q2/3-Q4^2^	n.s.
											
**Grade**	1	**5**	10.0	**0**	0.0	**2**	8.3	**3**	23.1	**Q1-Q2/3-Q4^1^**	***p *< 0.003**
	2	**24**	48.0	**2**	15.4	**15**	62.5	**7**	53.8	Q1-Q2/3^2^	*p *< 0.003
	3	**21**	42.0	**11**	84.6	**7**	29.2	**3**	23.1	Q1-Q4^2^	*p *< 0.003
										Q2/3-Q4^2^	n.s.
											
**Subgroups**	Luminal A	**25**	53.2	**1**	9.1	**18**	75.0	**6**	50.0	**Q1-Q2/3-Q4^1^**	***p *< 0.007**
	Luminal B	**6**	12.8	**3**	27.3	**0**	0.0	**3**	25.0	Q1-Q2/3^2^	*p *< 0.003
	ERBB2	**7**	14.9	**3**	27.3	**3**	12.5	**1**	8.3	Q1-Q4^2^	*p *< 0.05
	Basal	**4**	8.5	**2**	18.2	**1**	4.2	**1**	8.3	Q2/3-Q4^2^	n.s.
	Normal-like	**5**	10.6	**2**	18.2	**2**	8.3	**1**	8.3		

^1 ^for Q1-Q2/3-Q4 the Kruskal Wallis test was performed

^2 ^for Q1-Q2/3 the Mann-Whitney test was performed

^2 ^for Q1-Q4 the Mann-Whitney test was performed

^2 ^for Q2/3-Q4 the Mann-Whitney test was performed

**Table 4:** p53 or Δp53 mutant classes and their relation to molecular subgroups*

		Wild type (Wt)		Mutation group I (MI) missense and in frame mutations		Mutation group II (MII) nonsense, frame shift and splice mutations			
						
		No. of patients	(%)	No. of patients	(%)	No. of patients	(%)	Groups	*p*
**full-length p53**	Luminal A	**25**	53.2	**4**	15.4	**0**	0.0	**Wt-MI-MII^1^**	***p *< 0.006**
	Luminal B	**6**	12.8	**6**	23.1	**3**	27.3	Wt-MI^2^	*p *< 0.02
	ERBB2	**7**	14.9	**7**	26.9	**4**	36.4	Wt-MII^2^	*p *< 0.008
	Basal	**4**	8.5	**9**	34.6	**3**	27.3	MI-MII^2^	n.s.
	Normal-like	**5**	10.6	**0**	0.0	**1**	9.1	Wt-MI&MII^2^	*p *< 0.002
									
**Δp53**	Luminal A	**27**	46.6	**2**	13.3	**0**	0.0	**Wt-MI-MII^1^**	***p *< 0.04**
	Luminal B	**8**	13.8	**4**	26.7	**3**	27.3	Wt-MI^2^	n.s. (*p *< 0.07)
	ERBB2	**10**	17.2	**3**	20.0	**5**	45.5	Wt-MII^2^	*p *< 0.03
	Basal	**8**	13.8	**6**	40.0	**2**	18.2	MI-MII^2^	n.s.
	Normal-like	**5**	8.6	**0**	0.0	**1**	9.1		

^1 ^for WT-MI-MII the Kruskal Wallis test was performed

^2 ^for WT-MI the Mann-Whitney test was performed

* The tumors have previously been subjected to whole genome microarray analysis and classified into subgroups according to their expression profile (see Material and Methods)

## Discussion

Recently, several new p53 isoforms have been detected, but their functional roles, particular in tumorigenesis, remain unclear and require further investigations (see perspective [17]). Δp53 is one of these novel isoforms, it arises by an uncommon alternative splice mechanism, exhibits a p53-independent transcriptional activity, and a gene activation pattern different from that of p53 in cell lines [24]. Recently, it was shown that cells from patients with acute myeloid leukemia induction of chemotherapy modulates the p53/Δp53 protein ratio pattern [47].

In this study we have investigated Δp53 in-silico and at the mRNA expression level in relation to wild-type and mutated full-length p53 in order to determine possible correlations to biological and clinical parameters in human breast tumors. Our bioinformatic analysis revealed a high score for exonic splicing enhancers for the cassette sequence motif. We performed three-dimensional predictions for the structure of the Δp53 isoform, and confirmed its mRNA expression in human tumors. Although the model prediction identity for Δp53 was lower than for p53, we were able to identify that a major alpha-helical structure is missing at the C-terminal end, changing the further orientation of the protein resulting in a more compacted structure. These structural changes may explain the inability of Δp53 to form hetero-tetramers with full-length p53, and the different transcriptional activity of Δp53 independent from full-length p53 [24]. Activity differences have also been observed in other p53 isoforms: The Δ40p53 isoform is not activated in response to genotoxic stress [19], p53i9 is defective in transcriptional activity [18], and the p53AS isoform in mice displays different DNA-binding efficiencies [48].

The three-dimensional predictions and the functional analysis of Δp53 in cell lines encouraged us to investigate Δp53 function in relation to full-length p53 in human tumors. The special alternative splice process removes all mutations inside the spliced-out sequence and, as a consequence, the mutational statuses of the tumors are affected differentially for p53 than for Δp53. These "mutational hybrid" tumors have a mutated full-length p53 and a non-mutated Δp53. We investigated whether patients containing "mutational hybrid" tumors had biological and clinical parameters different from other types of mutations. No significant differences to specific changes in survival rate, in clinical or biological parameters were observed, although a slightly higher frequency of patients with distant metastasis at time of diagnosis was found. Thus, wild-type Δp53 does not seem to compensate for mutated p53, but possibly exerts adverse effects in tumors expressing mutant p53. One may speculate that a correct balance between full-length p53 and Δp53 is required to full-fill specific patterns in control of cell-cycle regulation and thus influences the overall survival in patients with a disturbed Δp53/p53 phenotype. Since this dataset is small, larger cohorts are necessary to confirm these findings.

Full-length p53 and Δp53 mRNA expression levels were measured by qRT-PCR. We could confirm that Δp53 mRNA is expressed in a mixture of different human cancer cell lines and in tumors, with Δp53 being expressed at a lower level than full-length p53. The mRNA levels of the two isoforms p53 and Δp53 are highly correlated. A 25–30% reduction in expression levels has also been reported for the mouse ASp53 isoform [49]. It may be a general feature that N- or C-terminal end truncated isoforms rather modulate p53 functions than abrogate it completely [16]. The existence of Δp53 mRNA is further confirmed by the observation that an in frame mutation located in the splice cassette led to high full-length p53 but reduced Δp53 mRNA expression, distinguishable from all others in frame or missense mutations. These and other results from different isoforms [22] form a picture according to which full-length p53 is the most highly expressed form during genotoxic stress.

In a series of 88 advanced primary breast tumors we investigated whether certain clinical parameters are related with the expression patterns of p53 and Δp53. It has previously been shown [22] that various p53 isoforms are expressed in human breast tumors, but correlation of expression levels to clinical data or mutational status was missing in that study. Steady-state amounts of mRNAs in genes related to breast cancer have very rarely been measured [50]. In the examined breast tumors we recognized for both p53 and Δp53 that the different mutation types show particular mRNA expression patterns. In comparison to wild-type mRNA expression, tumors with missense and in frame mutations had significantly increased amounts of mRNA, while in tumors with nonsense, frame shift and splice mutations mRNA levels were significantly reduced for both isoforms. Our study is thus one of the first confirming that mRNA expression of both the full-length p53 and the Δp53 form are elevated in tumors with missense and in frame mutations. The high level of p53 protein seen in mutated tumors has previously been explained by accumulation of the protein due to lack of degradation of the mutated protein and not by overexpression at the mRNA level. After DNA damage p53 is activated and Mdm2-p53 interaction decreases [4,51]. In a situation with increased levels of mutated p53, the disturbed dynamics of this fine-balance may result in an unsatisfied request for functional p53 activity inside the cell.

The molecular breast cancer subtypes [41,42] differ significantly with respect to frequencies of p53 mutations. The majority of wild-type tumors is classified as Luminal A subtype, while the majority of tumors with missense and in frame mutations belong to the Basal subtype and the ERBB2 subtype has mainly nonsense, frame shift or splice mutations. The different subgroups have different survival with the poorest survival rates for the class with highest p53 mutation rate [33,43,52].

We observed that the expression of full-length wild-type p53 was widely scattered, despite the significant differences between mutation groups as described above. To explore this unexpected result we divided the tumors into four p53 wild-type expression groups by quartiles. The tumors in the lowest quartile group were significantly associated with estrogen-negative receptor staining, high grade and the Luminal B and ERBB2 breast cancer subtypes, while tumors in the two middle quartiles showed significant association with a high fraction to estrogen receptor positive tumors, low grade and tumors of the Luminal A subtype. Tumors in the highest quartile of p53 abundance were associated with negative estrogen receptor status, low grade and the Luminal A breast cancer subtype. These findings are interesting and warrant further investigations in order to elucidate the molecular basis for these expression "extremes" in the wild-type *p53 *gene. Explanations may comprise technical reasons, like difficulties in detection of all types of mutations by standard techniques, or a biological basis due to mutations in genes other than p53 itself, disrupting the p53-signaling pathway. Future studies with an enlarged cohort size are required to determine whether the different distributions of low and high p53 mRNA level in the molecular subgroups are the cause or effect of the tumor.

## Conclusion

The tumor suppressor and transcription factor *p53 *is accompanied by different alternative splice forms. In silico analysis indicate three-dimensional and functional differences of the Δp53 and full-length p53 isoforms. Quantitative real-time PCR confirmed Δp53 mRNA expression with strong correlation between the two isoforms, however, with 2.64 higher levels for the p53 full-length form, both in mutated and non-mutated tumors. If at all, "mutational hybrid" patients had a slightly worse prognosis than patients with p53 mutations in both isoforms, indicating that wild-type Δp53 does not replace the p53 function lost by mutation, but rather might exert an adverse effect. The mRNA expression of p53 and Δp53 level showed a wide range in p53 wild-type tumors, with significant association to molecular breast cancer subtype distribution. In tumors, different mutation-dependent mRNA expression patterns were found with significant higher mRNA expression of both isoforms from missense or in frame *p53 *mutated genes compared to the wild-type *p53 *gene. A significant association was found for the distribution of breast cancer subtypes for wild-type and mutated Δp53 and the scattering of p53 mRNA expression levels revealed differences in wild-type p53 or mutated p53 tumors among the various subtypes.

## Materials and methods

### Patients

A total of 88 breast tumors samples from patients with advanced disease were selected from 4 different cohorts (A, B, C, and D): Fifty-six of the patients were part of a prospective study at the Haukeland University Hospital Bergen (Norway) on locally advanced breast cancer (T3/T4 and/or N2 tumors). Of these, thirty patients (A) received adjuvant doxorubicin monotherapy [44] and twenty-six patients (B) received adjuvant 5-fluorouracil and mitomycin treatment [45] before surgery. Twenty-three breast carcinoma specimens (C) were obtained from patients surgically treated at the National Cancer Institute of Milan (Italy) in 2002 ([53] and unpublished). Nine breast tumor samples (D) were from a series of patient samples sequentially collected at Ullevål University Hospital (Norway) from 1990–94 [54]. All breast carcinoma samples were frozen immediately after surgery and stored at -70°C to -80°C. Total RNA was isolated from snap frozen tumor tissue using TRIzol^® ^solution (Invitrogen™). The concentration of total RNA was determined using an HP 8453 spectrophotometer (Hewlett Packard) and the integrity of the RNA was assessed using a 2100 Bioanalyzer (Agilent) for series C and D.

### Mutation analysis

Mutation analysis of p53 was performed by pre-screening exon 2–11 using Temporal Temperature Gradient Gel Electrophoresis (TTGE), as described elsewhere [55], followed by sequencing. Previous mutation screening of these patients revealed 50 tumors with wild-type full-length p53, whereas in the other 38 tumors 25 missense mutations, 2 nonsense mutations, 6 frame shift, 2 in frame mutations and 3 splice mutations were detected ([33,44,45,53], for summary see Additional file 2).

### Biological and clinical factors

The estrogen receptor (ER) and progesterone receptor (PR) status were analyzed using both immunohistochemistry (IHC) and biochemical/ligand-binding assay (Abbott Diagnostics). Lymph node status, grade, and distant metastasis at time of diagnosis were available for patients in cohorts A, B and D, ERBB2/HER status were available for patients in cohorts A and D, and response to chemotherapy were available for patients in cohorts A and B [33,44,45]. For patients in cohort C, the lymph node status and histological grade were obtained from histological reports. Hormone receptors status and ERBB2/HER status were performed using IHC and scored using a semi-quantitative evaluation. Age, menopausal status, lymph node status, tumor histology, or p53 LOH were available for patients in all cohorts ([44,45,53,54] and unpublished). Breast cancer subtype classification was based on variation in gene expression derived from microarray experiments. Each sample was assigned to its subclass by the correlation to the centroid for the subclass [43]. Breast cancer subtype assignment for patients in the cohorts A, B, and D have previously been published [40-43] and are unpublished for patients in cohort C.

### Quantitative real time PCR (qRT-PCR)

#### RT and qPCR reaction

RT-reaction was performed using the GeneAmp^® ^RNA PCR kit from Applied Biosystems. A 20 μl reaction contained 4 μl 25 mM MgCl_2 _solution, 2 μl 10× PCR Buffer II, 1 μl H_2_O, premixed Deoxyribonucleoside triphospahtes: 2 μl dGTP, 2 μl dATP, 2 μl dTTP, 2 μl dCTP (10 mM each), 1 μl RNase Inhibitor (20 U/μl), 1 μl Random hexamers and 1 μl MuLV Reverse Transcriptase (50 U/μl) as a master mix, and 2 μl of total RNA was added prior to reaction start. Based on a previous photometric measurement the total RNA template concentration was below the reaction capacity of ≤1 μg RNA per reaction. Adapted times and temperature profiles for the reverse transcription were used:Incubation for 10 min at 25°C, 30 min at 42°C for reverse transcription of RNA, 5 min at 95°C for denaturation and 5 min at 5°C to cool down the reaction. For each series of cDNA reactions negative controls were added to insure contamination free consumables for the RT-reaction. After the RT-reaction samples were diluted 1:4 to get a final concentration of about 10 ng/μl cDNA.

#### qPCR reaction

qPCR reaction was done in a final volume of 25 μ containing 12,5 μl TaqMan^® ^Universal PCR Master Mix (Applied Biosystems, contains 2× AmpliTaq Gold^® ^DNA Polymerase, AmpErase^® ^UNG, dNTPs (with dUTP), Passive Reference 1, and optimized buffer components with proprietary formulation), 6,75 μl H_2_O, 1,25 μl (18 μM) forward primer, 1,25 μl (18 μM) reverse primers, and 1,25 μl (5 μM) probe (following producers recommended concentrations of 900 nM for primers and 250 nM for the probe). The reaction was put in an ABI PRISM^® ^96-Well Optical Reaction Plate placed on ice before 2 μl of the diluted cDNA template (approx. 10 ng/μl) was added. In a series of pre-experiments this concentration had turned out as the lowest amount required producing reliable and reproducible results in a reaction. The standard thermal cycling conditions of initial 50°C 2 min and 95°C 10 min followed by 40 cycles at 95°C for 15 s and 60°C for 1 min were used. All reactions were performed using an ABI PRISM^® ^7000 Sequence Detection System (TaqMan^®^). Experiments were performed in triplets for the standard curve and duplicates for all data points. Each qPCR reaction included no-template controls and five points of the standard curve in 1.5 orders of linear dynamic dilution range. For the standard curve a commercially available Universal Human Reference RNA (Stratagene, La Jolla, USA) was used consisting of equal amounts of RNA from 10 different human cancer cell lines. Analysis settings for threshold and the Comparative Ct (Cycle threshold) were set to auto and adjusted manually, if necessary.

#### Analytical Method

Briefly, in real time PCR the exponential increase in fluorescence signal during cycling is measured to generate a quantitative relation to the target amount at reaction start. Two analytical methods are established for qRT-PCR measurement, the standard curve method and the comparative Ct method (ΔΔCt). The benefit of the standard curve method is its independence of variations in amplification efficiencies between different genes, different splice variants, or between the target gene and the endogenous controls. The comparative Ct method was used to compare the linearized (2^-ΔΔCt^) expression levels of the standard curves of the two alternative splice forms relative to each other, whereas for the various tumor samples the more robust standard curve method was applied.

#### Primer and probes

Primers and probes for the p53 and Δp53 mRNA sequence were designed with the assistance of the Primer Express^® ^software (Applied Biosystems). We tested several primer pairs, highest specificity for Δp53 was reached with a forward primer covering exon 7, the splice cassette and the exon 9 junction. For the full-length p53 the forward primer covers the exon 6/7 junction, respectively. The sequence of the various primers were:

p53: TP53 C1S8-FP 5-GCCCCCAGGGAGCACTA-3;

TP53 C1S8-RP 5-GGGAGAGGAGCTGGTGTTG-3;

TP53 C1S8-PP 5-FAM-TTGGGCAGTGCTCGCT-MGB-3.

Δp53: TP53 E6/7c-FP 5-TGAGGTTGGCTCTGACTGTACC-3;

delta TP53 E7/9c-RP 5-CTCCATCCAGTGTGATGATGGT-3;

delta TP53-PP 5-FAM-GCAGGAACTGTTACACATG-MGB-3.

pmm1: PMM1-FP 5-ATCAACTTCTGCCTCAGCTACATG-3;

PMM1-RP 5-CCATTCCGGAACTCGATGA-3;

PMM1-PP 5-FAM-AGGTTCCACGCTTCT-MGB-3 (modified after [46]).

rpl32: RPL32-FP 5-ACCAGTCAGACCGATATGTCAAAA-3;

RPL32-RP 5-TTGTCAATGCCTCTGGGTTTC-3;

RPL32-PP 5-FAM-CGCCAGTTACGCTTAA-MGB-3 (modified after [46]).

#### Endogenous control

A general problem for gene expression measurements using qRT-PCR is to adjust for differences in loading, PCR inhibition or degradation. Photometric measurements are unreliable for sensible methods like qRT-PCR, as they do not take into account differences in the RT-reaction or changes in the mRNA/total RNA ratio. Many technical papers and reviews have addressed the problem of proper endogenous control genes for the normalization of qRT-PCR performance [46,56-61]. However, these studies show often contradictory results and do not come up with a "golden rule". We have carefully investigated most of the commonly used "housekeeping" genes, like the human 18S ribosomal RNA (18S), β-actin, cyclophilin, glyceraldehydes-3-phosphatase dehydrogenase (GAPDH), β2-microglobulin, β-glucronidase (GUS), hypoxanthine ribosyl transferase (HPRT), and the transcription factor IID TATA binding protein (TBP). We have rejected most of these commonly used endogenous controls based on the existence of retropseudogenes for some of these genes (β-actin, GADH, HPRT [62-64]) or on their previous observed tumor or cancer type specific expression alteration patterns (β-actin, GAPDH, TBP, cyclophilin [58,60,65-67]). Especially GAPDH correlates with clinical and molecular parameters in human breast cancer and should not be used as control RNA [68]. We followed the recommendation of [60] to use at least two endogenous controls. Based on the investigations of [46,59,61], and our own survey of these genes in microarray experiments, we choose the two endogenous controls LP32 and PMM1 for this study. The traditional loading control 18S rRNA was included as reference to previous studies and thus, low, medium and highly expression ranges were covered by our endogenous controls. However, 18S rRNA is overexpressed in mammary gland and colon cancer [66,69], affected by various biological factors and drugs [70,71], and shows an imbalance between mRNA and the rRNA content in mammalian mammary tumors [72]. Comparison of expression variation in our study showed that the highest divergence was found for 18S and consequently it was omitted. All relative mRNA quantity values of p53 and Δp53 were normalized to the average levels of the two independent endogenous control references PMM1 and RPL32.

### Software applications and statistical analysis

p53 protein domain classification and their locations along the protein were specified according to Swiss-Prot/TrEMBL [12]. Exonic splicing enhancers (ESEs) binding sites for splicing factors of the serine/arginine-rich (SR) protein family were analyzed using the ESEfinder software [36]. For three-dimensional structural prediction the complete sequences of p53 and Δp53 were submitted to the CPHmodels 2.0 server [73], a web-based CPH-modeling service of the Technical University of Denmark [39]. The bits scores of the model prediction indicate the precision of a match to a HMM profile. Alignments scores are commonly reported as bits scores: The likelihood that the query sequence is a plausible homologue of the database sequence is compared to the likelihood that the sequence was instead generated by a "random" model. The log2 of this likelihood ratio gives the bits score. The three-dimensional structure prediction models were illustrated using the RasMol program (Win Molecular Graphics Windows Version 2.7.2.1.1.). For survival and all other statistical analysis the software package SPSS^® ^for Windows (Release 12.0.2, 24 Mar 2004; Copyright ^®^SPSS Inc.) was used. Differences between mutant groups, in clinical or molecular parameters were analyzed using the Kruskal Wallis rank tests for k independent samples and the Mann-Whitney test for independent association analysis between any two subgroups. Breast cancer survival was analyzed by the log-rank test and illustrated as Kaplan-Meier plots.

## Competing interests

The author(s) declare no financial or non-financial competing interests.

## Authors' contributions

**LOB **carried out the structural in silico analysis and the qRT-PCR studies including primer design and analysis; LOB further performed all data analysis, writing of the manuscript, including preparations of all figures and tables.

**SM **carried out parts of the qRT-PCR studies.

**AL **carried out RNA preparation, p53 mutation analysis and provision of molecular and clinical data for Ullevål University Hospital cohort.

**AB **carried out RNA preparation, p53 mutation analysis and provision of molecular and clinical data for National Cancer Institute of Milan cohort.

**SBG **carried out tumor preparation and provision of molecular and clinical data for doxorubicin and 5-fluorouracil and mitomycin cohorts from the Haukeland University Hospital Bergen.

**PEL **was responsible for collection of material and lab performance at the Haukeland University Hospital Bergen, critical reading of the draft.

**WD **suggested this study and participated in its design, critical reading of the manuscript.

**ID **discovered the Δp53 isoform, provided its sequence and cell line based data for approval in human breast tumors, critical reading of the draft.

**A-LB-D **conceived the study, and participated in its design and coordination and helped to draft the manuscript.

All authors read and approved the final manuscript.

## Accession Numbers

***p53 ***[EMBL: AF307851, HGNC: 11998, PIR: A25224, Swiss-Prot: P04637, RefSeq: NM_000546, Ensembl: ENSG00000141510].

***pmm1 ***(phosphomannomutase 1) [NCBI: U86070, D87810, HSU86070, SwissProt: Q9287, RefSeq: NM_002676, Ensembl: ENSG00000100417].

***rpl32 ***(ribosomal protein L32) [NCBI: X03342, SwissProt: P62910, RefSeq: NM_000994, Ensembl: ENSG00000144713].

## Supplementary Material

Additional File 1 **mRNA and aa sequence of p53 and Δp53**. p53 mRNA and its translated protein sequence. The untranslated precursor and untranslated region afterwards are illustrated with yellow highlights. Alternating exons are written in consecutive black and blue and translation codon triplets are marked with alternating white and light yellow. The removed alternative splice sequence of Δp53 is shown with light blue colour and the alternative splice cassettes are indicated with red. Sequence information is based on ENSEMBL notification [74]. Click here for file

Additional File 2 **p53 and Δp53 mutation specifications**. This table lists the coded patient sample IDs, mutation classifications for p53 and Δp53 (including codon, nucleotides and aa changes), incidents of flagging primers and the numeric qRT-PCR expression levels. Click here for file

Additional File 3 **Relationship between Δp53 status and the standard clinical, pathological and biological factors**. The data provided represent the relationship between Δp53 status and the standard clinical, pathological and biological factors. Click here for file

## References

[B1] Kim E Deppert W The complex interactions of p53 with target DNA: we learn as we go Biochem Cell Biol 2003 81 141 150 10.1139/o03-046 12897847

[B2] May P May E Twenty years of p53 research: structural and functional aspects of the p53 protein Oncogene 1999 18 7621 7636 10.1038/sj.onc.1203285 10618702

[B3] Sengupta S Harris CC p53: traffic cop at the crossroads of DNA repair and recombination Nat Rev Mol Cell Biol 2005 6 44 55 10.1038/nrm1546 15688066

[B4] Vogelstein B Lane D Levine AJ Surfing the p53 network Nature 2000 408 307 310 10.1038/35042675 11099028

[B5] el Deiry WS Regulation of p53 downstream genes Semin Cancer Biol 1998 8 345 357 10.1006/scbi.1998.0097 10101800

[B6] Guimaraes DP Hainaut P TP53: a key gene in human cancer Biochimie 2002 84 83 93 10.1016/S0300-9084(01)01356-6 11900880

[B7] Wahl GM Linke SP Paulson TG Huang LC Maintaining genetic stability through TP53 mediated checkpoint control Cancer Surv 1997 29 183 219 9338102

[B8] Hainaut P Wiman K Eds 25 Years of p53 Research 2005 Dordrecht, The Netherlands: Springer

[B9] Soussi T Caron dF May P Structural aspects of the p53 protein in relation to gene evolution Oncogene 1990 5 945 952 2142762

[B10] Kim E Deppert W Transcriptional activities of mutant p53: when mutations are more than a loss J Cell Biochem 2004 93 878 886 10.1002/jcb.20271 15449312

[B11] Kim E Deppert W The versatile interactions of p53 with DNA: when flexibility serves specificity Cell Death Differ 2006 13 885 889 10.1038/sj.cdd.4401909 16543936

[B12] Swiss-Prot/TrEMBL Feature aligner http://www.expasy.org/cgi-bin/aligner?P04637

[B13] el Deiry WS Kern SE Pietenpol JA Kinzler KW Vogelstein B Definition of a consensus binding site for p53 Nat Genet 1992 1 45 49 10.1038/ng0492-45 1301998

[B14] Jeffrey PD Gorina S Pavletich NP Crystal structure of the tetramerization domain of the p53 tumor suppressor at 1.7 angstroms Science 1995 267 1498 1502 10.1126/science.7878469 7878469

[B15] Kussie PH Gorina S Marechal V Elenbaas B Moreau J Levine AJ Pavletich NP Structure of the MDM2 oncoprotein bound to the p53 tumor suppressor transactivation domain [comment] Science 1996 274 948 953 10.1126/science.274.5289.948 8875929

[B16] Courtois S de Fromentel CC Hainaut P p53 protein variants: structural and functional similarities with p63 and p73 isoforms Oncogene 2004 23 631 638 10.1038/sj.onc.1206929 14737098

[B17] Mills AA p53: link to the past, bridge to the future Genes Dev 2005 19 2091 2099 10.1101/gad.1362905 16166374

[B18] Flaman JM Waridel F Estreicher A Vannier A Limacher JM Gilbert D Iggo R Frebourg T The human tumour suppressor gene p53 is alternatively spliced in normal cells Oncogene 1996 12 813 818 8632903

[B19] Courtois S Verhaegh G North S Luciani MG Lassus P Hibner U Oren M Hainaut P DeltaN-p53, a natural isoform of p53 lacking the first transactivation domain, counteracts growth suppression by wild-type p53 Oncogene 2002 21 6722 6728 10.1038/sj.onc.1205874 12360399

[B20] Ghosh A Stewart D Matlashewski G Regulation of human p53 activity and cell localization by alternative splicing Mol Cell Biol 2004 24 7987 7997 10.1128/MCB.24.18.7987-7997.2004 15340061 PMC515058

[B21] Yin Y Stephen CW Luciani MG Fahraeus R p53 Stability and activity is regulated by Mdm2-mediated induction of alternative p53 translation products Nat Cell Biol 2002 4 462 467 10.1038/ncb801 12032546

[B22] Bourdon JC Fernandes K Murray-Zmijewski F Liu G Diot A Xirodimas DP Saville MK Lane DP p53 isoforms can regulate p53 transcriptional activity Genes Dev 2005 19 2122 2137 10.1101/gad.1339905 16131611 PMC1221884

[B23] Matlashewski G Pim D Banks L Crawford L Alternative splicing of human p53 transcripts Oncogene Res 1987 1 77 85 2453013

[B24] Rohaly G Chemnitz J Dehde S Nunez AM Heukeshoven J Deppert W Dornreiter I A novel human p53 isoform is an essential element of the ATR-intra-S phase checkpoint Cell 2005 122 21 32 10.1016/j.cell.2005.04.032 16009130

[B25] Prives C Manfredi JJ The continuing saga of p53–more sleepless nights ahead Mol Cell 2005 19 719 721 10.1016/j.molcel.2005.08.024 16168367

[B26] Caron dF Soussi T TP53 tumor suppressor gene: a model for investigating human mutagenesis Genes Chromosomes Cancer 1992 4 1 15 1377002 10.1002/gcc.2870040102

[B27] Hollstein M Rice K Greenblatt MS Soussi T Fuchs R Sorlie T Hovig E Smith-Sorensen B Montesano R Harris CC Database of p53 gene somatic mutations in human tumors and cell lines Nucleic Acids Res 1994 22 3551 3555 7937055 PMC308317

[B28] Børresen-Dale AL TP53 and breast cancer Hum Mutat 2003 21 292 300 10.1002/humu.10174 12619115

[B29] Hollstein M Hergenhahn M Yang Q Bartsch H Wang ZQ Hainaut P New approaches to understanding p53 gene tumor mutation spectra Mutat Res 1999 431 199 209 10635987 10.1016/s0027-5107(99)00162-1

[B30] Olivier M Hainaut P TP53 mutation patterns in breast cancers: searching for clues of environmental carcinogenesis Semin Cancer Biol 2001 11 353 360 10.1006/scbi.2001.0390 11562177

[B31] Olivier M Eeles R Hollstein M Khan MA Harris CC Hainaut P The IARC TP53 database: new online mutation analysis and recommendations to users Hum Mutat 2002 19 607 614 10.1002/humu.10081 12007217

[B32] Walker DR Bond JP Tarone RE Harris CC Makalowski W Boguski MS Greenblatt MS Evolutionary conservation and somatic mutation hotspot maps of p53: correlation with p53 protein structural and functional features Oncogene 1999 18 211 218 10.1038/sj.onc.1202298 9926936

[B33] Olivier M Langerod A Carrieri P Bergh J Klaar S Eyfjord J Theillet C Rodriguez C Lidereau R Bieche I Varley J Bignon Y Uhrhammer N Winqvist R Jukkola-Vuorinen A Niederacher D Kato S Ishioka C Hainaut P Borresen-Dale AL The clinical value of somatic TP53 gene mutations in 1,794 patients with breast cancer Clin Cancer Res 2006 12 1157 1167 10.1158/1078-0432.CCR-05-1029 16489069

[B34] Overgaard J Yilmaz M Guldberg P Hansen LL Alsner J TP53 mutation is an independent prognostic marker for poor outcome in both node-negative and node-positive breast cancer Acta Oncol 2000 39 327 333 10.1080/028418600750013096 10987229

[B35] van Oijen MG Slootweg PJ Gain-of-function mutations in the tumor suppressor gene p53 Clin Cancer Res 2000 6 2138 2145 10873062

[B36] Cartegni L Wang J Zhu Z Zhang MQ Krainer AR ESEfinder: A web resource to identify exonic splicing enhancers Nucleic Acids Res 2003 31 3568 3571 10.1093/nar/gkg616 12824367 PMC169022

[B37] Ladd AN Cooper TA Finding signals that regulate alternative splicing in the post-genomic era Genome Biol 2002 3 reviews0008.1 0008.16 10.1186/gb-2002-3-11-reviews0008 12429065 PMC244920

[B38] PROSITE documentation http://expasy.org/prosite/PDOC00015

[B39] Lund O Nielsen M Lundegaard P Worning P CPHmodels 2.0: X3M a Computer Program to Extract 3D Models [abstract] CASP5 conference 2002

[B40] Langerod A Molecular Profiling of Breast Cancer – From single gene variants to whole genome expression patterns PhD thesis 2005 University of Oslo, Faculty of Medicine

[B41] Perou CM Sorlie T Eisen MB van de RM Jeffrey SS Rees CA Pollack JR Ross DT Johnsen H Akslen LA Fluge O Pergamenschikov A Williams C Zhu SX Lonning PE Borresen-Dale AL Brown PO Botstein D Molecular portraits of human breast tumours Nature 2000 406 747 752 10.1038/35021093 10963602

[B42] Sorlie T Perou CM Tibshirani R Aas T Geisler S Johnsen H Hastie T Eisen MB van de RM Jeffrey SS Thorsen T Quist H Matese JC Brown PO Botstein D Eystein LP Borresen-Dale AL Gene expression patterns of breast carcinomas distinguish tumor subclasses with clinical implications Proc Natl Acad Sci USA 2001 98 10869 10874 10.1073/pnas.191367098 11553815 PMC58566

[B43] Sorlie T Tibshirani R Parker J Hastie T Marron JS Nobel A Deng S Johnsen H Pesich R Geisler S Demeter J Perou CM Lonning PE Brown PO Borresen-Dale AL Botstein D Repeated observation of breast tumor subtypes in independent gene expression data sets Proc Natl Acad Sci USA 2003 100 8418 8423 10.1073/pnas.0932692100 12829800 PMC166244

[B44] Geisler S Lonning PE Aas T Johnsen H Fluge O Haugen DF Lillehaug JR Akslen LA Borresen-Dale AL Influence of TP53 gene alterations and c-erbB-2 expression on the response to treatment with doxorubicin in locally advanced breast cancer Cancer Res 2001 61 2505 2512 11289122

[B45] Geisler S Borresen-Dale AL Johnsen H Aas T Geisler J Akslen LA Anker G Lonning PE TP53 gene mutations predict the response to neoadjuvant treatment with 5-fluorouracil and mitomycin in locally advanced breast cancer Clin Cancer Res 2003 9 5582 5588 14654539

[B46] Hamalainen HK Tubman JC Vikman S Kyrola T Ylikoski E Warrington JA Lahesmaa R Identification and validation of endogenous reference genes for expression profiling of T helper cell differentiation by quantitative real-time RT-PCR Anal Biochem 2001 299 63 70 10.1006/abio.2001.5369 11726185

[B47] Anensen N Oyan AM Bourdon JC Kalland KH Bruserud O Gjertsen BT A distinct p53 protein isoform signature reflects the onset of induction chemotherapy for acute myeloid leukemia Clin Cancer Res 2006 12 3985 3992 10.1158/1078-0432.CCR-05-1970 16818696

[B48] Kulesz-Martin MF Lisafeld B Huang H Kisiel ND Lee L Endogenous p53 protein generated from wild-type alternatively spliced p53 RNA in mouse epidermal cells Mol Cell Biol 1994 14 1698 1708 8114705 10.1128/mcb.14.3.1698-1708.1994 PMC358528

[B49] Han KA Kulesz-Martin MF Alternatively spliced p53 RNA in transformed and normal cells of different tissue types Nucleic Acids Res 1992 20 1979 1981 1579500 10.1093/nar/20.8.1979 PMC312315

[B50] Perrin-Vidoz L Sinilnikova OM Stoppa-Lyonnet D Lenoir GM Mazoyer S The nonsense-mediated mRNA decay pathway triggers degradation of most BRCA1 mRNAs bearing premature termination codons Hum Mol Genet 2002 11 2805 2814 10.1093/hmg/11.23.2805 12393792

[B51] Lahav G Rosenfeld N Sigal A Geva-Zatorsky N Levine AJ Elowitz MB Alon U Dynamics of the p53-Mdm2 feedback loop in individual cells Nat Genet 2004 36 147 150 10.1038/ng1293 14730303

[B52] Miller LD Smeds J George J Vega VB Vergara L Ploner A Pawitan Y Hall P Klaar S Liu ET Bergh J An expression signature for p53 status in human breast cancer predicts mutation status, transcriptional effects, and patient survival Proc Natl Acad Sci USA 2005 102 13550 13555 10.1073/pnas.0506230102 16141321 PMC1197273

[B53] Kringen P Bergamaschi A Due EU Wang Y Tagliabue E Nesland JM Nehman A Tonisson N Borresen-Dale AL Evaluation of arrayed primer extension for TP53 mutation detection in breast and ovarian carcinomas Biotechniques 2005 39 755 761 16312222 10.2144/000112000

[B54] Bukholm IK Nesland JM Karesen R Jacobsen U Borresen AL Relationship between abnormal p53 protein and failure to express p21 protein in human breast carcinomas J Pathol 1997 181 140 145 10.1002/(SICI)1096-9896(199702)181:2<140::AID-PATH745>3.0.CO;2-A 9120716

[B55] Sorlie T Johnsen H Vu P Lind GE Lothe R Borresen-Dale AL Keohavong P, Grant SG Mutation screening of the TP53 gene by temporal temperature gradient gel electrophoresis Methods in Molecular Biology 2005 291 Totowa, NJ: Humana Press Inc 207 216 15502225 10.1385/1-59259-840-4:207

[B56] Dheda K Huggett JF Bustin SA Johnson MA Rook G Zumla A Validation of housekeeping genes for normalizing RNA expression in real-time PCR Biotechniques 2004 37 112 119 15283208 10.2144/04371RR03

[B57] Lossos IS Czerwinski DK Wechser MA Levy R Optimization of quantitative real-time RT-PCR parameters for the study of lymphoid malignancies Leukemia 2003 17 789 795 10.1038/sj.leu.2402880 12682639

[B58] Schmittgen TD Zakrajsek BA Effect of experimental treatment on housekeeping gene expression: validation by real-time, quantitative RT-PCR J Biochem Biophys Methods 2000 46 69 81 10.1016/S0165-022X(00)00129-9 11086195

[B59] Thellin O Zorzi W Lakaye B De Borman B Coumans B Hennen G Grisar T Igout A Heinen E Housekeeping genes as internal standards: use and limits J Biotechnol 1999 75 291 295 10.1016/S0168-1656(99)00163-7 10617337

[B60] Vandesompele J De Preter K Pattyn F Poppe B Van Roy N De Paepe A Speleman F Accurate normalization of real-time quantitative RT-PCR data by geometric averaging of multiple internal control genes Genome Biol 2002 3 RESEARCH0034 10.1186/gb-2002-3-7-research0034 12184808 PMC126239

[B61] Warrington JA Nair A Mahadevappa M Tsyganskaya M Comparison of human adult and fetal expression and identification of 535 housekeeping/maintenance genes Physiol Genomics 2000 2 143 147 11015593 10.1152/physiolgenomics.2000.2.3.143

[B62] Bieche I Laurendeau I Tozlu S Olivi M Vidaud D Lidereau R Vidaud M Quantitation of MYC gene expression in sporadic breast tumors with a real-time reverse transcription-PCR assay Cancer Res 1999 59 2759 2765 10383126

[B63] Gibson UE Heid CA Williams PM A novel method for real time quantitative RT-PCR Genome Res 1996 6 995 1001 8908519 10.1101/gr.6.10.995

[B64] Sellner LN Turbett GR The presence of a pseudogene may affect the use of HPRT as an endogenous mRNA control in RT-PCR Mol Cell Probes 1996 10 481 483 10.1006/mcpr.1996.0068 9025089

[B65] de Kok JB Roelofs RW Giesendorf BA Pennings JL Waas ET Feuth T Swinkels DW Span PN Normalization of gene expression measurements in tumor tissues: comparison of 13 endogenous control genes Lab Invest 2005 85 154 159 15543203 10.1038/labinvest.3700208

[B66] Gerard CJ Andrejka LM Macina RA Mitochondrial ATP synthase 6 as an endogenous control in the quantitative RT-PCR analysis of clinical cancer samples Mol Diagn 2000 5 39 46 10.1016/S1084-8592(00)00009-6 10837088

[B67] White RJ RNA polymerase III transcription and cancer Oncogene 2004 23 3208 3216 10.1038/sj.onc.1207547 15094770

[B68] Revillion F Pawlowski V Hornez L Peyrat JP Glyceraldehyde-3-phosphate dehydrogenase gene expression in human breast cancer Eur J Cancer 2000 36 1038 1042 10.1016/S0959-8049(00)00051-4 10885609

[B69] Mullen CA Review: analogies between trophoblastic and malignant cells Am J Reprod Immunol 1998 39 41 49 9458933 10.1111/j.1600-0897.1998.tb00332.x

[B70] Spanakis E Problems related to the interpretation of autoradiographic data on gene expression using common constitutive transcripts as controls Nucleic Acids Res 1993 21 3809 3819 8367299 10.1093/nar/21.16.3809 PMC309896

[B71] Warner JR The economics of ribosome biosynthesis in yeast Trends Biochem Sci 1999 24 437 440 10.1016/S0968-0004(99)01460-7 10542411

[B72] Solanas M Moral R Escrich E Unsuitability of using ribosomal RNA as loading control for Northern blot analyses related to the imbalance between messenger and ribosomal RNA content in rat mammary tumors Anal Biochem 2001 288 99 102 10.1006/abio.2000.4889 11141312

[B73] CPHmodels 2.0 Server http://www.cbs.dtu.dk/services/CPHmodels/

[B74] Ensembl Transcript Report http://www.ensembl.org/Homo_sapiens/transview?transcript=ENST00000269305&db=core&show=peptide&number=on

