# Supplementary material for: Expression of full-length p53 and its isoform Δp53 in breast carcinomas in relation to mutation status and clinical parameters
Source: Mol Cancer. 2006 Oct 20;5:47. doi: 10.1186/1476-4598-5-47 (PMC1636663; doi:10.1186/1476-4598-5-47)
Supplement: Additional File 1 — mRNA and aa sequence of p53 and Δp53. p53 mRNA and its translated protein sequence. The untranslated precursor and untranslated region afterwards are illustrated with yellow highlights. Alternating exons are written in consecutive black and blue and translation codon triplets are marked with alternating white and light yellow. The removed alternative splice sequence of Δp53 is shown with light blue colour and the alternative splice cassettes are indicated with red. Sequence information is based on ENSEMBL notification [74]. [file 1476-4598-5-47-S1.pdf]

Additional file 1: mRNA and aa sequence of p53 and Δp53

```
   1 ACTTGTCATGGCGACTGTCCAGCTTTGTGCCAGGAGCCTCGCAGGGGTTGATGGGATTGG
     ............................................................
  61 GGTTTTCCCCTCCCATGTGCTCAAGACTGGCGCTAAAAGTTTTGAGCTTCTCAAAAGTCT
     ............................................................
 121 AGAGCCACCGTCCAGGGAGCAGGTAGCTGCTGGGCTCCGGGGACACTTTGCGTTCGGGCT
     ............................................................
 181 GGGAGCGTGCTTTCCACGACGGTGACACGCTTCCCTGGATTGGCAGCCAGACTGCCTTCC
     ............................................................
 241 GGGTCACTGCCATGGAGGAGCCGCAGTCAGATCCTAGCGTCGAGCCCCCTCTGAGTCAGG
     ...........-M--E--E--P--Q--S--D--P--S--V--E--P--P--L--S--Q--
 301 AAACATTTTCAGACCTATGGAAACTACTTCCTGAAAACAACGTTCTGTCCCCCTTGCCGT
  17 E--T--F--S--D--L--W--K--L--L--P--E--N--N--V--L--S--P--L--P--
 361 CCCAAGCAATGGATGATTTGATGCTGTCCCCGGACGATATTGAACAATGGTTCACTGAAG
  37 S--Q--A--M--D--D--L--M--L--S--P--D--D--I--E--Q--W--F--T--E--
 421 ACCCAGGTCCAGATGAAGCTCCCAGAATGCCAGAGGCTGCTCCCCCCGTGGCCCCTGCAC
  57 D--P--G--P--D--E--A--P--R--M--P--E--A--A--P--P--V--A--P--A--
 481 CAGCAGCTCCTACACCGGCGGCCCCTGCACCAGCCCCCTCCTGGCCCCTGTCATCTTCTG
  77 P--A--A--P--T--P--A--A--P--A--P--A--P--S--W--P--L--S--S--S--
 541 TCCCTTCCCAGAAAACCTACCAGGGCAGCTACGGTTTCCGTCTGGGCTTCTTGCATTCTG
  97 V--P--S--Q--K--T--Y--Q--G--S--Y--G--F--R--L--G--F--L--H--S--
 601 GGACAGCCAAGTCTGTGACTTGCACGTACTCCCCTGCCCTCAACAAGATGTTTTGCCAAC
 117 G--T--A--K--S--V--T--C--T--Y--S--P--A--L--N--K--M--F--C--Q--
 661 TGGCCAAGACCTGCCCTGTGCAGCTGTGGGTTGATTCCACACCCCCGCCCGGCACCCGCG
 137 L--A--K--T--C--P--V--Q--L--W--V--D--S--T--P--P--P--G--T--R--
 721 TCCGCGCCATGGCCATCTACAAGCAGTCACAGCACATGACGGAGGTTGTGAGGCGCTGCC
 157 V--R--A--M--A--I--Y--K--Q--S--Q--H--M--T--E--V--V--R--R--C--
 781 CCCACCATGAGCGCTGCTCAGATAGCGATGGTCTGGCCCCTCCTCAGCATCTTATCCGAG
 177 P--H--H--E--R--C--S--D--S--D--G--L--A--P--P--Q--H--L--I--R--
 841 TGGAAGGAAATTTGCGTGTGGAGTATTTGGATGACAGAAACACTTTTCGACATAGTGTGG
 197 V--E--G--N--L--R--V--E--Y--L--D--D--R--N--T--F--R--H--S--V--
 901 TGGTGCCCTATGAGCCGCCTGAGGTTGGCTCTGACTGTACCACCATCCACTACAACTACA
 217 V--V--P--Y--E--P--P--E--V--G--S--D--C--T--T--I--H--Y--N--Y--
 961 TGTGTAACAGTTCCTGCATGGGCGGCATGAACCGGAGGCCCATCCTCACCATCATCACAC
 237 M--C--N--S--S--C--M--G--G--M--N--R--R--P--I--L--T--I--I--T--
1021 TGGAAGACTCCAGTGGTAATCTACTGGGACGGAACAGCTTTGAGGTGCGTGTTTGTGCCT
 257 L--E--D--S--S--G--N--L--L--G--R--N--S--F--E--V--R--V--C--A--
1081 GTCCTGGGAGAGACCGGCGCACAGAGGAAGAGAATCTCCGCAAGAAAGGGGAGCCTCACC
 277 C--P--G--R--D--R--R--T--E--E--E--N--L--R--K--K--G--E--P--H--
1141 ACGAGCTGCCCCCAGGGAGCACTAAGCGAGCACTGCCCAACAACACCAGCTCCTCTCCCC
 297 H--E--L--P--P--G--S--T--K--R--A--L--P--N--N--T--S--S--S--P--
1201 AGCCAAAGAAGAAACCACTGGATGGAGAATATTTCACCCTTCAGATCCGTGGGCGTGAGC
 317 Q--P--K--K--K--P--L--D--G--E--Y--F--T--L--Q--I--R--G--R--E--
1261 GCTTCGAGATGTTCCGAGAGCTGAATGAGGCCTTGGAACTCAAGGATGCCCAGGCTGGGA
 337 R--F--E--M--F--R--E--L--N--E--A--L--E--L--K--D--A--Q--A--G--
1321 AGGAGCCAGGGGGGAGCAGGGCTCACTCCAGCCACCTGAAGTCCAAAAAGGGTCAGTCTA
 357 K--E--P--G--G--S--R--A--H--S--S--H--L--K--S--K--K--G--Q--S--
1381 CCTCCCGCCATAAAAAACTCATGTTCAAGACAGAAGGGCCTGACTCAGACTGACATTCTC
 377 T--S--R--H--K--K--L--M--F--K--T--E--G--P--D--S--D--*-.......
1441 CACTTCTTGTTCCCCACTGACAGCCTCCCACCCCCATCTCTCCCTCCCCTGCCATTTTGG
     ............................................................
1501 (…) to 2621
```
