# Supplementary material for: Expression of full-length p53 and its isoform Δp53 in breast carcinomas in relation to mutation status and clinical parameters
Source: Mol Cancer. 2006 Oct 20;5:47. doi: 10.1186/1476-4598-5-47 (PMC1636663; doi:10.1186/1476-4598-5-47)
Supplement: Additional File 2 — p53 and Δp53 mutation specifications. This table lists the coded patient sample IDs, mutation classifications for p53 and Δp53 (including codon, nucleotides and aa changes), incidents of flagging primers and the numeric qRT-PCR expression levels. [file 1476-4598-5-47-S2.pdf]

Additional file 2: p53 and Δp53 mutation specifications

ID (coded)	p53 mutation class	Δp53 mutation class	p53/Δp53 mutation class	p53 codon	nucleotide change	aa change	flagging primers	p53 exp (a.u.)	Δp53 exp (a.u.)
DO103A	0	0	0	-	-	-	0	1.286	1.031
DO104A	0	0	0	-	-	-	0	0.826	0.891
DO106B	0	0	0	-	-	-	0	0.866	0.881
DO116A	0	0	0	-	-	-	0	1.003	1.167
DO117A	0	0	0	213	CGA->CGG	pm, arg->arg	0	0.601	0.595
DO118B	0	0	0	-	-	-	0	0.859	0.932
DO119A	0	0	0	-	-	-	0	0.067	0.115
DO121B	0	0	0	-	-	-	0	0.857	0.790
DO124A	0	0	0	-	-	-	0	1.099	1.215
DO214B	0	0	0	-	-	-	0	0.242	0.382
DO308B	0	0	0	-	-	-	0	1.038	1.057
DO402B	0	0	0	-	-	-	0	0.943	1.046
DO702B	0	0	0	-	-	-	0	1.414	1.467
DO710A	0	0	0	-	-	-	0	1.278	1.199
DO807A	0	0	0	-	-	-	0	0.502	0.724
FU01	0	0	0	-	-	-	0	0.422	0.390
FU02	0	0	0	-	-	-	0	0.759	0.653
FU09	0	0	0	33 (before)	C->A 29 bp før ex4	pm	0	0.523	0.542
FU10	0	0	0	-	-	-	0	0.849	0.801
FU14	0	0	0	-	-	-	0	2.037	1.346
FU15	0	0	0	-	-	-	0	1.114	0.862
FU22	0	0	0	-	-	-	0	0.810	0.699
FU24	0	0	0	-	-	-	0	0.508	0.467
FU25	0	0	0	-	-	-	0	0.748	0.507
FU35	0	0	0	-	-		0	0.734	0.666
FU37	0	0	0	-	-	-	0	0.898	0.810
FU39	0	0	0	-	-	-	0	1.286	1.189
M011	0	0	0	-	-	-	0	0.426	0.343
M012	0	0	0	-	-	-	0	0.450	0.289
M014	0	0	0	-	-	-	0	0.319	0.286
M020	0	0	0	-	-	-	0	0.453	0.378
M024	0	0	0	-	-	-	0	0.313	0.276
M036	0	0	0	36	g->a (108)	pm, pro->pro	0	0.687	0.567
M076	0	0	0	-	-	-	0	0.583	0.482
M091	0	0	0	-	-	-	0	0.759	0.666
M140	0	0	0	-	-	-	0	0.117	0.104
M164	0	0	0	213	>g (639)	pm, arg->arg	0	0.218	0.191
M185	0	0	0	213	>g (639)	pm, arg->arg	0	0.774	0.548
M191	0	0	0	-	-	-	0	0.280	0.202
M235	0	0	0	-	-	-	0	1.820	1.306
M346	0	0	0	-	-	-	0	1.108	0.974
M350	0	0	0	-	-	-	0	0.390	0.332
M387	0	0	0	-	-	-	0	0.596	0.509
SM217	0	0	0	-	-	-	0	0.494	0.536
ULL014	0	0	0	-	-	-	0	0.586	0.512
ULL022	0	0	0	-	-	-	0	1.016	0.801
ULL046	0	0	0	-	-	-	0	0.630	0.926
ULL066	0	0	0	-	-	-	0	1.353	1.200
ULL169	0	0	0	-	-	-	0	0.307	0.362
ULL176	0	0	0	-	-	-	0	1.245	1.121
DO107B	1	0	1	273	CGT->CAT	arg->his	0	0.650	0.725
DO120A	1	0	1	266	GGA->AGA	gly->arg	0	0.953	1.036
DO208A	1	0	1	273	CGT->CAT	arg->his	0	2.454	2.334
DO404B	1	0	1	286	GAA->AAA	glu->cys	0	2.090	2.407
DO405A	1	0	1	273	CGT->CAT	arg->his	0	1.846	1.767
DO706A	1	0	1	277	TGT->TAT	cys->tyr	0	1.650	1.538
FU12	1	0	1	282	CGG->GGG	arg->gly	0	0.832	0.752
FU26	1	0	1	273	CGT->CCT	arg->pro	0	1.073	0.902
M136	1	0	1	278, 213	c->g (832); >g (639)	pro->arg; arg->arg	0	0.383	0.261
M259	1	0	1	275	g->t (824)	cys->phe	0	1.159	0.909
DO111B	1	1	2	249	AGG->GGG	arg->gly	0	1.612	1.578
DO115B	1	1	2	163	TAC->TGC	tyr->cys	0	1.985	2.351
DO205A	1	1	2	176	TGC->TTC	cys->phe	0	1.575	1.572
DO206A	1	1	2	237	ATG->ATT	met->ile	Δp53 prim	2.231	-
DO605B	1	1	2	175	CGC->CAC	arg->his	0	1.022	0.974
FU05	1	1	2	195	ATC->ACC	ile->thr	0	1.536	1.380
FU20	1	1	2	220	TAT->TGT	tyr->cys	0	2.394	2.019
FU23	1	1	2	195	ATC->ACC	ile->thr	0	1.515	1.480
FU30	1	1	2	163	TAC->TGC	tyr ->cys	0	0.979	0.843
FU49	1	1	2	135	TGC->TAC	cys->tyr	0	0.908	0.741
M059	1	1	2	179	c->t (535)	his->tyr	0	0.827	0.650
M347	1	1	2	241	c->g (722)	ser->cys	Δp53 prim	0.739	0.268
ULL080	1	1	2	143	GTG > ATG	val->met	0	0.522	0.640
ULL177	1	1	2	110	CGT > CCT	arg->pro	0	0.585	0.639
ULL202	1	1	2	163	TAC > CAC	tyr ->his	0	0.663	0.514
FU06	2	0	1	298 (stop at 298)	GAG->TAG	glu->stop	p53 prim	0.232	0.229
M151	2	2	3	166 (stop at 166)	c->a (497)	ser->stop	0	0.192	0.295
DO123B	3	3	3	217-221 (stop 223/224)	14 bp del (217-221)		0	0.220	0.247
DO305A	3	3	3	239-242 (stop 262/263)	11 bp del (239-242)		Δp53 prim	0.959	0.862
FU11	3	3	3	140-143 (stop 169)	10 bp del (140-143)		0	0.827	0.616
FU19	3	3	3	108 (stop 147)	2 bp del GG		0	0.357	0.310
M345	3	3	3	178 (stop ?)	5 bp del (533-538)		0	0.204	0.221
FU08	3	6	3	308 - 331 (stop 335/336)	74 bp, insert dupl. ex9		p53 prim	0.627	0.548
DO303B	4	4	2	233-235	6 bp del (233-235)		0	1.648	1.914
FU27	4	6	2	256	12 bp ins		Δp53 prim	1.527	0.302
DO105A	5	5	3	126-132 (cDNA)	g->a; 1 bp før exon 5, 21bp del 126-132(cDNA)		0	1.083	1.296
FU04	5	5	3	after 331 (unsecure)	g->c; 1 bp downstream ex 9		0	0.504	0.362
FU07	5	5	3	187-224; (unsecure)	t->g; 3 bp upstream ex 6		0	0.249	0.247

p53 mutation	Δp53 mutation	p53-Δp53 relation
0 = Wt (and pm)	0 = Wt Δp53	0 = Wtp53 + WtΔp53
1 = missence	1 = missence	1 = M p53 + WtΔp53
2 = nonsence	2 = nonsence	2 = MI p53 + MI Δp53
3 = frame shift	3 = frame shift	3 = MII p53 + MII Δp53
4 = in frame	4 = in frame	
5 = splice	5 = splice	
	6 = in splice cassette	

flagging primers

Δp53 prim = mutation location in Δp53 primer binding site
p53 prim = mutation location in p53 primer binding site

"-" = missing value
due to technical reasons
(a.u. = arbitrary units)
