# Supplementary material for: Expression of full-length p53 and its isoform Δp53 in breast carcinomas in relation to mutation status and clinical parameters
Source: Mol Cancer. 2006 Oct 20;5:47. doi: 10.1186/1476-4598-5-47 (PMC1636663; doi:10.1186/1476-4598-5-47)
Supplement: Additional File 3 — Relationship between Δp53 status and the standard clinical, pathological and biological factors. The data provided represent the relationship between Δp53 status and the standard clinical, pathological and biological factors. [file 1476-4598-5-47-S3.pdf]

Additional file 3: Relationship between Δp53 status and the standard clinical, pathological and biological factors

		**Δp53**							
		**Wild type (Wt)**		**Mutation group I (MI) Mutations: MS, IF**		**Mutation group II (MII) Mutations: NS, FS, SP**			
**Characteristic**		**No. of patients (%)**		**No. of patients (%)**		**No. of patients (%)**		**Groups**	***p***
**Lymph node status**	Node-negative	**17**	28.3	**3**	18.8	**7**	63.6	**Wt-MI-MII[1]**	**$p<0.04$**
	Node-positive	**43**	71.7	**13**	81.3	**4**	36.4	Wt-MI[2]	n.s.
								Wt-MII[2]	$p<0.03$
								MI-MII[2]	$p<0.02$
**Estrogen receptor status**	Negative	**13**	21.3	**8**	50.0	**3**	27.3	**Wt-MI-MII[1]**	**n.s. ($p<0.08$)**
	Positive	**48**	78.7	**8**	50.0	**8**	72.7	Wt-MI[2]	$p<0.03$
								Wt-MII[2]	n.s.
								MI-MII[2]	n.s.
**Progesterone receptor status**	Negative	**17**	27.9	**8**	50.0	**7**	63.6	**Wt-MI-MII[1]**	**$p<0.04$**
	Positive	**44**	72.1	**8**	50.0	**4**	36.4	Wt-MI[2]	n.s. ($p<0.1$)
								Wt-MII[2]	$p<0.03$
								MI-MII[2]	n.s.
**ERBB2/HER status**	Negative	**34**	77.3	**10**	90.9	**1**	20.0	**Wt-MI-MII[1]**	**$p<0.009$**
	Positive	**10**	22.7	**1**	9.1	**4**	80.0	Wt-MI[2]	n.s.
								Wt-MII[2]	$p<0.008$
								MI-MII[2]	$p<0.006$
**Distant metastisis at time of diagnosis**	Negative	**37**	88.1	**14**	100.0	**8**	88.9	**Wt-MI-MII[1]**	**n.s.**
	Positive	**5**	11.9	**0**	0.0	**1**	11.1	Wt-MI[2]	n.s.
								Wt-MII[2]	n.s.
								MI-MII[2]	n.s.
**Grade**	1	**5**	8.2	**0**	0.0	**0**	0.0	**Wt-MI-MII[1]**	**n.s. ($p<0.07$)**
	2	**28**	45.9	**4**	25.0	**4**	36.4	Wt-MI[2]	$p<0.04$
	3	**28**	45.9	**12**	75.0	**7**	63.6	Wt-MII[2]	n.s.
								MI-MII[2]	n.s.
**LOH P53**	heterozygot	**13**	34.2	**0**	0.0	**1**	14.3	**Wt-MI-MII[1]**	**n.s. ($p<0.06$)**
	allelic imbalance	**25**	65.8	**11**	100.0	**6**	85.7	Wt-MI[2]	$p<0.03$
								Wt-MII[2]	n.s.
								MI-MII[2]	n.s.
**Subgroups**	Lum A	**27**	46.6	**2**	13.3	**0**	0.0	**Wt-MI-MII[1]**	**$p<0.04$**
	Lum B	**8**	13.8	**4**	26.7	**3**	27.3	Wt-MI[2]	n.s. ($p<0.07$)
	ERBB2	**10**	17.2	**3**	20.0	**5**	45.5	Wt-MII[2]	$p<0.03$
	Basal	**8**	13.8	**6**	40.0	**2**	18.2	MI-MII[2]	n.s.
	Normal	**5**	8.6	**0**	0.0	**1**	9.1		

[1] for WT-MI-MII the Kruskal Wallis test was performed
[2] for WT-MI the Mann-Whitney test was performed
[2] for WT-MII the Mann-Whitney test was performed
[2] for MI-MII the Mann-Whitney test was performed
